# Ultrasonography Comparison of Pelvic Floor and Abdominal Wall Muscles in Women with and without Dyspareunia: A Cross-Sectional Study

**DOI:** 10.3390/diagnostics12081827

**Published:** 2022-07-29

**Authors:** Elena Castellanos-López, Camila Castillo-Merino, Vanesa Abuín-Porras, Daniel López-López, Carlos Romero-Morales

**Affiliations:** 1 Faculty of Sport Sciences, Universidad Europea de Madrid, C/Tajo s/n, Villaviciosa de Odón, 28670 Madrid, Spain; e.castellanoslopezas@gmail.com (E.C.-L.); camilacastillomerino@gmail.com (C.C.-M.); carlos.romero@universidadeuropea.es (C.R.-M.); 2 Research, Health and Podiatry Group, Department of Health Sciences, Faculty of Nursing and Podiatry, Industrial Campus of Ferrol, Universidade da Coruña, 15403 Ferrol, Spain; daniel.lopez.lopez@udc.es

**Keywords:** physiotherapy, dyspareunia, pelvic floor, abdominal muscles, ultrasound

## Abstract

Pelvic floor hypertonicity and narrowing of the levator ani hiatus is traditionally assumed in women with dyspareunia and considered a therapeutical target by physical therapists. However, accurate pre-treatment assessment of pelvic floor muscles is difficult to perform in clinical sites. In addition, the abdominal musculature has not been evaluated in this population, despite its relationship with pelvic floor disfunctions. The purpose of this study was to determine the existence of differences in the length of the anteroposterior diameter of the levator ani hiatus (APDH), the thickness of the abdominal wall musculature and the interrecti distance (IRD) in subjects with dyspareunia compared to a control group. A cross-sectional observational study was designed using ultrasound imaging to measure the APH, the thickness of the abdominal musculature—rectus abdominis (RA), transverse abdominis (TrAb), internal oblique (IO), external oblique (EO)—and IRD at rest and during contraction. Thirty-two women were recruited through advertising and social webs and divided into two groups: dyspareunia (n = 16) and no dyspareunia (n = 16). There were no statistically significant differences (*p* < 0.05) in RA, TrAb, OI and OE muscle thickness. No differences in APH or in supraumbilical and infraumbilical IRD were found. The findings of this study suggest that the relationship between the abdominal structure/levator ani hypertonia and dyspareunia remains uncertain.

## 1. Introduction

Dyspareunia (DP) is a condition defined by the onset of abdominal, pelvic or vaginal pain during or after vaginal penetration and/or coitus [1]. Women affected by DP report difficulties in vaginal penetration associated with pain, fear, anxiety and involuntary contraction of pelvic floor muscles [2]. This condition is classified by the location of pain (entry or deep DP) or based on the onset (primary or secondary) [3]. Prevalence of DP is difficult to stablish, due to cultural factors that cause woman to hide their pain [4]. The reported prevalence in some countries ranges from 8% to 54.5% [4,5].

Physical therapy treatment of DP generally includes several modalities, involving electrical stimulation, physical agents (i.e., heat or cold) and manual techniques, aiming to reduce pelvic floor muscle hypertonia. Nevertheless, there is lack of specificity on the targeted areas. This inaccuracy can be justified by the complexity and multifactorial nature of DP, in which different superficial and deep pelvic floor muscles present hypertonia and weakness simultaneously [5]. Several studies have included surface electromyography for specific assessment [6], but this form of evaluation is not commonly available in daily clinical practice.

Ultrasound imaging (USI) has been reported in scientific literature as a safe and reliable method to assess pelvic floor and abdominal wall muscles, through three different approaches: transabdominal, introital and transperineal [7,8,9,10]. Previous USI assessment of the anteroposterior diameter of the levator ani hiatus (APDH) found a correlation with levator ani distensibility. Moreover, this distensibility is associated with the appropriate function of the pelvic floor biomechanics. Narrowing of this APDH has been related to hypertonia [9,11,12,13]. USI measures of the APDH have been used to evaluate pelvic floor function in different statuses (rest, active contraction and Valsalva) and to assess pre-post treatment changes [14].

The abdominal wall creates a synergy with pelvic floor muscles as a part of the deep stabilization system of the trunk [15]. In addition, the activation of the abdominal wall muscles is related to an increase in the abdominal pressure [16] and changes in the pelvic floor [17], such as increasing the contraction of the bladder neck muscle [18]. Interrecti distance (IRD) in women has been addressed by several authors. An increased IRD, commonly found in woman in their postpartum period, could be a predictor of pelvic floor disorders such as urinary incontinence [19]. Moreover, some authors have assessed the abdominal wall muscle thickness and IRD in other pain-related gynecological condition, dysmenorrhea, with discrepancy in their results [20,21]. Therefore, further studies are needed about the role of the central nervous system processing in painful conditions and its relation to pelvic muscle imbalance [21].

Considerations in scientific literature about muscle architecture in women with DP are limited. However, some authors report hypertonia and higher reflex responses in superficial pelvic floor muscles in women with DP. Deep muscle involvement still remains unclear [22,23,24]. Several studies conducted in gynecological cancer survivors and patients with endometriosis showed differences in pelvic floor architecture related with DP [9,24,25,26]. APH and hiatus area have been explored in several studies in women after childbirth, concluding that no direct or isolated relation with DP could be found [14,27]. To the authors’ knowledge, abdominal wall features of this group have not been assessed up to this date.

The purpose of this study was to determinate, through USI assessment, whether differences in APH, abdominal wall muscle thickness and IRD could be found in two groups of women, with and without DP. Our hypothesis was that the DP group would present an increased APH and IRD and decreased abdominal wall thickness.

## 2. Materials and Methods

### 2.1. Study Design

A cross-sectional, observational, case–control study was designed following the STROBE (Strengthening the Reporting of Observational Studies in Epidemiology) guidelines [28].

### 2.2. Ethical Considerations

The ethics committee of Universidad Europea de Madrid approved the present study (CIPI/22.012). It also adhered to the ethical standards of the Declaration of Helsinki. Informed consent was obtained from the participants before inclusion.

### 2.3. Participants

**Figure 1:**
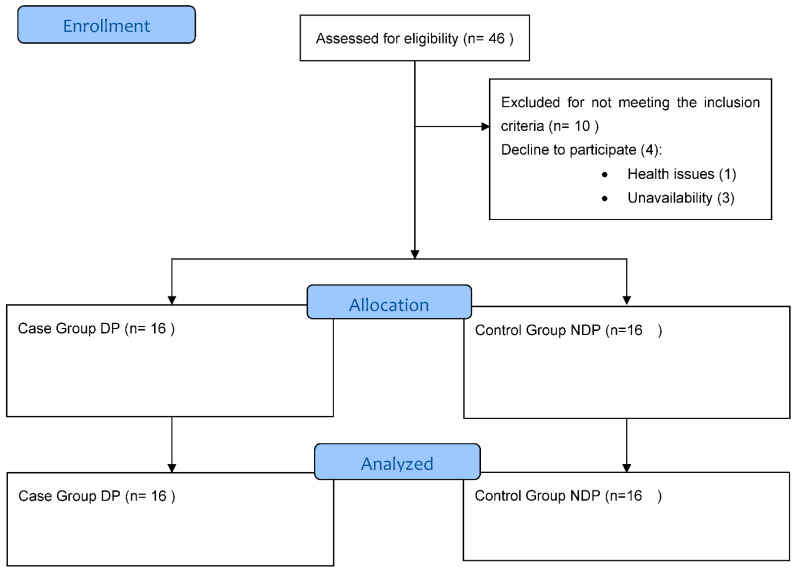
Flow chart.

In total, 32 subjects were recruited through social webs, including social webs, advertising through physical therapy clinic sites and promotion of the study between university students. Potential participants were asked to complete a simple online questionnaire in order to perform a pre-screening. If they self-reported dyspareunia symptoms, they were assigned to the case group, consecutively, and the same for the control group. The sample was divided into two groups: dyspareunia (DP) (n = 16) and no dyspareunia (NDP) (n = 16), matched by age quotes. The inclusion criteria were: (a) women between 18 and 45 years and (b) self-reported as sexually active in the pre-screening questionnaire. The exclusion criteria were: (a) IMC greater than 30 kg/m^2^ [29]; (b) pregnant [30]; (c) women in the postpartum period (6 months) [30]; (d) undergone abdominal surgery in the last 6 months; (e) significant musculoskeletal alterations that could interfere with the measurement [31]; (f) respiratory or neurological conditions [29]; and (g) presence of red flags [32,33]. For the DP group, DSM-5 criteria for definition of the symptoms were observed. The flow chart of the study can be observed in Figure 1. The facilities and lab instruments were provided by the European University of Madrid.

### 2.4. Sample Size Calculation

Sample size calculation was carried out by the difference between 2 independent groups with G*Power 3.1.9.2 software and based on the superior IRD at rest (cm) of a pilot study (*n* = 20) with 2 groups (mean ± SD): 10 DP (1.4 ± 0.36 cm) and 10 NDP (1.1 ± 0.23 cm). For the sample size calculation, 1-tailed hypothesis, an effect size of 0.99, an α error probability of 0.05, a power (1-β error probability) of 0.80, and an allocation ratio (N2/N1) of 1 were utilized. Thus, a total sample size of 28 subjects, 14 for each group, was calculated. Considering the 15% of possible participants lost to follow-up, a total sample of 32 subjects, 16 in each group, was considered. A post hoc power analysis was performed for the IRD variable, obtaining a 0.62 value.

### 2.5. Outcome Measures

**Figure 2:**
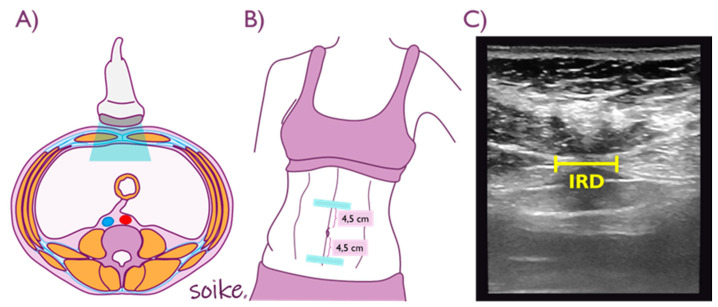
IRD assessment procedure. (**A**,**B**) Probe positioning. (**C**) Ultrasound image Source: Elena Castellanos López (AKA Soike).

All measures were performed with a high-quality ultrasound system (LOGIC F6, GE Healthcare, Chicago, IL, USA) with a lineal probe (frequency range 6 to 13 MHz) in B mode. Measurement was performed by the same evaluator, an expert in ultrasound imaging with several specialization courses and 5 years of experience in the use of USI. Every participant was placed in the supine position, with hip and knee flexion and a pillow supporting their heads. The Chiarello et al. protocol was followed for the IRD measurement, at two points, supraumbilical and infraumbilical (IRD SUP and IRD INF) (52). Measures were taken at rest and during the contraction of the rectus abdominis (RA), 4.5 cm above and 4.5 cm below the umbilicus (Figure 2). To achieve abdominal contraction, subjects were asked to cross their arms over the chest and raise their upper trunk until the spine of the scapulae had no contact with the surface. Inspiration or expiration state was not considered relevant for this measurement [34].

**Figure 3:**
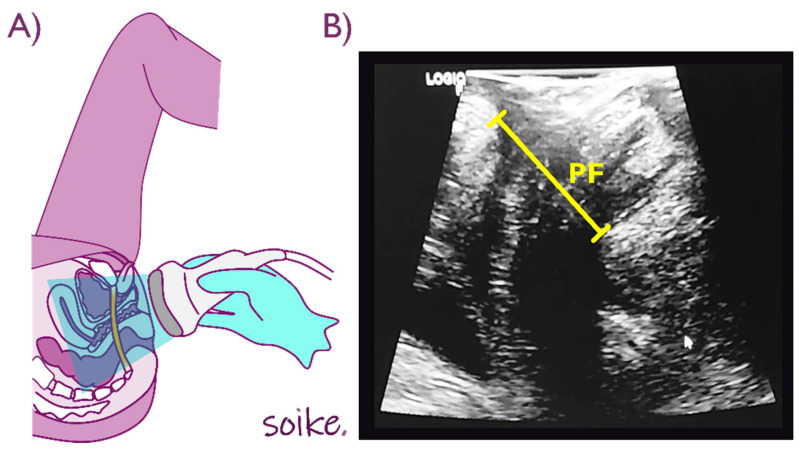
(**A**) Probe positioning, (**B**) pelvic floor (PF) muscle imaging. Source: Elena Castellanos López (AKA Soike).

Whittaker et al. [7] guidelines were followed for the USI assessment of the abdominal wall muscles. For the RA evaluation, the probe was placed immediately above the umbilicus and laterally from the midline, until the cross-sectional area of the muscle was centered in the image, at rest and during voluntary contraction. Images of the transversus abdominis (TrA), internal oblique (IO) and external oblique (EO) were taken with the probe placed transversally in the right side of the abdomen, between the edge of the eleventh rib and the iliac crest, at rest and during contraction. The Wen et al. [35] protocol was followed for the measurement of the APDH, ensuring that the participants had an empty bladder before the assessment. APDH was measured between the inferior border of the pubic symphysis and the anterior border of the pubovisceral muscle (Figure 3).

### 2.6. Statistical Analysis

Data resulting from the study were analyzed using the statistical software SPSS v.25 (IBM SPSS version 23.0, Chicago, IL, USA). Throughout the study, we used an α error of 0.05, a β error of 0.2 and a confidence interval of 95%. Descriptive analysis for the total sample and for each group was carried out. Parametric data were described using mean and standard deviation (SD), whereas non-parametric data were described using median and interquartile range (IR). Shapiro–Wilk test was performed to assess normality and the Levene test was used to assess the homogeneity of variances. For the comparative analysis of parametric data for both groups, the Student’s *t*-test for the independent samples was used, whereas for non-parametric data analysis, the Mann–Whitney *U* test was used. Intraclass correlation coefficients for evaluation of intra-rater reliability of the APDH measurement were reported. Values less than 0.4, between 0.4 and 0.55, between 0.56 and 0.75 and between 0.76 and 1 are indicative of poor, moderate, good and excellent reliability, respectively. The effect size between groups was estimated through the use of Cohens’s *d*, interpreting values of 0.2 as small, 0.5 as medium and 0.8 as large effects.

## 3. Results

Measures of age, weight and BMI were homogeneous between the DP and the NDP group. Nevertheless, there were statistically significant differences in height (*p* = 0.04; TE/d Cohen = −0.76, 95% CI) (Table 1).

No significant differences were found between the DP and NDP groups for TrA, IO, EO and IRD at contraction and rest time (*p* > 0.05; 95% CI) (Table 2). Moreover, ICC for APDH at rest (CCI = 0.98) and during voluntary contraction (CCI = 0.95) showed excellent intra-rater reliability.

## 4. Discussion

The aim of the present study was to determine whether there are differences in APDH, abdominal wall thickness and IRD supra and infraumbilical in woman with DP compared to women without DP. The results of the present study did not reveal statistically significant differences in any of the measures.

These results are coincident with Thibault-Gagnon et al. [36], who, in a sample of women affected by vestibulodynia—a type of DP characterized by pain in the vulvar vestibule—found no significant differences in APDH diameter compared to the controls. Moreover, the authors concluded that the interaction between deep and superficial pelvic floor muscles is complex, and APDH maybe has a stronger correlation with deep pelvic floor muscles, which seem to have a weaker relation with DP than superficial muscles. The aim of this study was to explore the specific relation of the APDH with DP, in order to establish noninvasive, rapid assessment tools for clinical practice. Therefore, the results of the present study support these conclusions, as there were no differences in APDH between groups. It must be pointed out that our inclusion criteria did not find differences between vestibulodynia and other DP types. Further research should be conducted to assess the involvement of different pelvic floor muscles related to DP classification. In addition, the exclusion criteria for this study did not contemplate the presence of endometriosis, that could be a confounder factor for dyspareunia [37,38,39]. Yong [40] pointed out in his study that it is necessary to explore comorbid conditions and central sensitization in DP, which could have acted as biases in the present study. Central sensitization is defined as a dysregulation of pain mechanisms in the central nervous system, that processes normal stimulus as painful [41,42,43]. Nevertheless, there are several authors with certain discrepancies in their results compared to ours. Cyr et al. [24] and Huffman et al. [25] observed, in gynecological cancer survivors, a narrowing of the hiatal area in DP subjects. However, hiatal area depends not only on the APDH, but also on the transverse diameter, which was not assessed in the present study. This could account for the discrepancy between the results and could guide clinical assessment to more accurate targets. Physical therapy treatments demonstrated positive results in the alterations of the hiatal area [24,25,26], so previous assessment through noninvasive methods, such as USI, could imply a positive impact in enhancing the effectiveness of the interventions.

Finally, no differences between abdominal wall muscle thickness and IRD were found between the two groups. There is a close interaction between the muscles and pelvic floor [15,44,45], so this could also explain the absence of differences in APDH between groups. Further studies comparing groups with increased IRD and hiatal area parameters related to DP could potentially clarify the extent of this interaction.

The present study presented several limitations. First, the probe used in the protocol was lineal instead of convex. Nevertheless, the configuration of the equipment allowed enough deep imaging to correctly identify the targeted area. In this study, only APDH was measured, which can also be a limitation. In addition, height was not homogeneous between groups. To the author’s knowledge, this variable has no direct influence on PF muscles, but the results should be considered according to this heterogeneity. It also had to be considered that the inspiration or exhalation state was not considered relevant for IRD measurement. Dyspareunia treatment has traditionally been focused on pain, whereas other difficulties associated with genito pelvic pain/penetration disorder (GGPPD) had been disregarded [40,46,47]. Future research lines should include physical aspects related to GGPPD, such as adherences, narrowing or involuntary contraction of the pelvic floor muscles. Finally, it is important to report that some women declined participation due to previous negative experiences in gynecological healthcare units. Traditional assessment includes many methods that could be perceived as invasive, such as electromyography or digital exploration, amongst others. Generalization of the use of noninvasive evaluation assessments could be of use to overcome this population’s reticence to search for treatment. Noninvasive, accurate assessment methods, such as USI, could facilitate the definition of “treatment target areas”, which would facilitate the improvement of gynecological physical therapy techniques.

## 5. Conclusions

The findings of the present study suggest that there is no relationship between the abdominal wall muscles/levator ani hypertonia and DP. Ultrasound assessment of the pelvic floor muscles could be considered as a noninvasive, reliable method to improve accuracy in therapeutic targets.

## Figures and Tables

**Table 1:** Sociodemographic data of the sample.

Data	DP (*n* = 16)	NDP (*n* = 16)	*p*-Value Cases vs. Controls
Age, years	25.38 ± 3.4 *	26.75 ± 3.99 *	0.303 **
Weight, kg	56.66 ± 10.36 *	62.69 ± 7.24 *	0.066 **
Height, m	1.61 ± 0.06 *	1.66 ± 0.06 *	0.04 **
BMI, kg/m^2^	21.74 ± 2.86 *	22.78 ± 1.70 *	0.221 **

Abbreviations: DP, dyspareunia. NDP, no dyspareunia. Body mass index (BMI). * Mean ± (standard deviation). ** Student’s *t*-test for independent samples was performed.

**Table 2:** Ultrasound imaging of the abdominal wall and pelvic floor muscles.

Measurement	DP (*n* = 16)	NDP (*n* = 16)	*p*-Value
**Distance (cm)**			
IRD SUP rest	0.93 ± 0.48 *^†^	1.3 ± 0.69 *	0.149 ^‡^
IRD SUP contraction	0.87 ± 0.5 *	1.14 ± 0.61 *	0.173 **
IRD INF rest	0.15 ± 0.15 *	0.21 ± 0.14 *	0.212 **
IRD SUP contraction	0.18 ± 0.16	0.22 ± 0.15 *	0.428 **
**Thickness (cm)**			
RA rest	0.93 ± 0.16 *	0.93 ± 0.14 *	0.958 **
RA contraction	1.17 ± 0.18 *	1.21 ± 0.25 *	0.624 **
TrAb rest	0.26 ± 0.06 *	0.28 ± 0.06 *	0.477 **
TrAb contraction	0.46 ± 0.11 *	0.46 ± 0.14 *	0.989 **
IO rest	0.49 ± 0.1 *	0.55 ± 0.12 *	0.131 **
IO contraction	0.59 ± 0.17 *	0.65 ± 0.15 *	0.27 **
EO rest	0.35 ± 0.07 *	0.39 ± 0.11 *	0.243 **
EO contraction	0.48 ± 0.15 *	0.52 ± 0.17 *	0.482 **
**APH (cm)**			
Rest	3.75 ± 0.57 *	3.66 ± 0.5 *	0.635 **
Contraction	3.29 ± 0.46 *	3.24 ± 0.35 *	0.723 **

Abbreviations: EO, external oblique; IO, internal oblique; IRD, interrecti distance; SUP, supraumbilical; INF, infraumbilical; RA, rectus anterior; TrAb, transversus abdominis; APDH, anteroposterior diameter hiatus; * Mean (standard deviation) was applied. ** Student´s *t*-test for independent samples was performed. ^†^ Median (25th percentile, 75th percentile) was used. ^‡^ Mann–Whitney U test was utilized.

## References

[1] Wallace S.L., Miller L.D., Mishra K. (2019). Pelvic floor physical therapy in the treatment of pelvic floor dysfunction in women. Curr. Opin. Obstet. Gynecol..

[2] Berghmans B. (2018). Physiotherapy for pelvic pain and female sexual dysfunction: An untapped resource. Int. Urogynecol. J..

[3] Alimi Y., Iwanaga J., Oskouian R.J., Loukas M., Tubbs R.S. (2018). The clinical anatomy of dyspareunia: A review. Clin. Anat..

[4] Laumann E.O., Paik A., Rosen R.C. (1999). Sexual Dysfunction in the United StatesPrevalence and Predictors. JAMA.

[5] Ghaderi F., Bastani P., Hajebrahimi S., Jafarabadi M.A., Berghmans B. (2019). Pelvic floor rehabilitation in the treatment of women with dyspareunia: A randomized controlled clinical trial. Int. Urogynecol. J..

[6] Morin M., Binik Y.M., Bourbonnais D., Khalifé S., Ouellet S., Bergeron S. (2017). Heightened Pelvic Floor Muscle Tone and Altered Contractility in Women with Provoked Vestibulodynia. J. Sex. Med..

[7] Whittaker J.L. (2008). Ultrasound imaging of the lateral abdominal wall muscles in individuals with lumbopelvic pain and signs of concurrent hypocapnia. Man. Ther..

[8] Whittaker J.L., Warner M.B., Stokes M. (2013). Comparison of the sonographic features of the abdominal wall muscles and connective tissues in individuals with and without lumbopelvic pain. J. Orthop. Sports Phys. Ther..

[9] Mabrouk M., Del Forno S., Spezzano A., Raimondo D., Arena A., Zanello M., Leonardi D., Paradisi R., Seracchioli R. (2020). Painful Love: Superficial Dyspareunia and Three Dimensional Transperineal Ultrasound Evaluation of Pelvic Floor Muscle in Women with Endometriosis. J. Sex Marital Ther..

[10] Morin M., Bergeron S., Khalifé S., Mayrand M.-H., Binik Y.M. (2014). Morphometry of the pelvic floor muscles in women with and without provoked vestibulodynia using 4D ultrasound. J. Sex. Med..

[11] Delancey J.O., Hurd W.W. (1998). Size of the urogenital hiatus in the levator ani muscles in normal women and women with pelvic organ prolapse. Obstet. Gynecol..

[12] Dietz H.P., Shek C., Clarke B. (2005). Biometry of the pubovisceral muscle and levator hiatus by three-dimensional pelvic floor ultrasound. Ultrasound Obstet. Gynecol. Off. J. Int. Soc. Ultrasound Obstet. Gynecol..

[13] Dietz H.P., Shek C., De Leon J., Steensma A.B. (2008). Ballooning of the levator hiatus. Ultrasound Obstet. Gynecol. Off. J. Int. Soc. Ultrasound Obstet. Gynecol..

[14] Roos A.-M., Speksnijder L., Steensma A.B. (2020). Postpartum sexual function; the importance of the levator ani muscle. Int. Urogynecol. J..

[15] Hsu S.L., Oda H., Shirahata S., Watanabe M., Sasaki M. (2018). Effects of neuromuscular training on core stability. J. Phys. Ther. Sci..

[16] Abuín-Porras V., Maldonado-Tello P., de la Cueva-Reguera M., Rodríguez-Sanz D., Calvo-Lobo C., López-López D., Navarro-Flores E., Romero-Morales C. (2020). Comparison of Lateral Abdominal Musculature Activation during Expiration with an Expiratory Flow Control Device Versus the Abdominal Drawing-in Maneuver in Healthy Women: A Cross-Sectional Observational Pilot Study. Medicina.

[17] Sapsford R.R., Hodges P.W., Richardson C.A., Cooper D.H., Markwell S.J., Jull G.A. (2001). Co-activation of the abdominal and pelvic floor muscles during voluntary exercises. Neurourol. Urodyn..

[18] Junginger B., Baessler K., Sapsford R., Hodges P.W. (2010). Effect of abdominal and pelvic floor tasks on muscle activity, abdominal pressure and bladder neck. Int. Urogynecol. J..

[19] Benjamin D.R., Frawley H.C., Shields N., van de Water A.T.M., Taylor N.F. (2019). Relationship between diastasis of the rectus abdominis muscle (DRAM) and musculoskeletal dysfunctions, pain and quality of life: A systematic review. Physiotherapy.

[20] Payne L.A., Seidman L.C., Sim M.S., Rapkin A.J., Naliboff B.D., Zeltzer L.K. (2019). Experimental evaluation of central pain processes in young women with primary dysmenorrhea. Pain.

[21] Romero-Morales C., de la Cueva-Reguera M., Miñambres-Vallejo B., Ruiz-Ruiz B., Calvo-Lobo C., Casado-Hernández I., López-López D., Abuín-Porras V. (2020). Ultrasound Assessment of the Abdominal Wall Muscles in Women with and without Primary Dysmenorrhea: A Cross-Sectional Study. Diagnostics.

[22] Rochera M.B. (2016). Physiotherapy in Treating Sexual Pain Disorders in Women: A Systematic Review. Adv. Sex. Med..

[23] Li-Yun-Fong R.J., Larouche M., Hyakutake M., Koenig N., Lovatt C., Geoffrion R., Brotto L.A., Lee T., Cundiff G.W. (2017). Is Pelvic Floor Dysfunction an Independent Threat to Sexual Function? A Cross-Sectional Study in Women with Pelvic Floor Dysfunction. J. Sex. Med..

[24] Cyr M.-P., Dumoulin C., Bessette P., Pina A., Gotlieb W.H., Lapointe-Milot K., Morin M. (2021). Characterizing Pelvic Floor Muscle Function and Morphometry in Survivors of Gynecological Cancer Who Have Dyspareunia: A Comparative Cross-Sectional Study. Phys. Ther..

[25] Huffman L.B., Hartenbach E.M., Carter J., Rash J.K., Kushner D.M. (2016). Maintaining sexual health throughout gynecologic cancer survivorship: A comprehensive review and clinical guide. Gynecol. Oncol..

[26] Del Forno S., Arena A., Pellizzone V., Lenzi J., Raimondo D., Cocchi L., Paradisi R., Youssef A., Casadio P., Seracchioli R. (2021). Assessment of levator hiatal area using 3D/4D transperineal ultrasound in women with deep infiltrating endometriosis and superficial dyspareunia treated with pelvic floor muscle physiotherapy: Randomized controlled trial. Ultrasound Obstet. Gynecol. Off. J. Int. Soc. Ultrasound Obstet. Gynecol..

[27] Falkert A., Willmann A., Endress E., Meint P., Seelbach-Göbel B. (2013). Three-dimensional ultrasound of pelvic floor: Is there a correlation with delivery mode and persisting pelvic floor disorders 18-24 months after first delivery?. Ultrasound Obstet. Gynecol. Off. J. Int. Soc. Ultrasound Obstet. Gynecol..

[28] von Elm E., Altman D.G., Egger M., Pocock S.J., Gøtzsche P.C., Vandenbroucke J.P. (2008). The Strengthening the Reporting of Observational Studies in Epidemiology (STROBE) statement: Guidelines for reporting observational studies. J. Clin. Epidemiol..

[29] Stetts D.M., Freund J.E., Allison S.C., Carpenter G. (2009). A rehabilitative ultrasound imaging investigation of lateral abdominal muscle thickness in healthy aging adults. J. Geriatr. Phys. Ther..

[30] van Veelen G.A., Schweitzer K.J., van der Vaart C.H. (2014). Ultrasound imaging of the pelvic floor: Changes in anatomy during and after first pregnancy. Ultrasound Obstet. Gynecol. Off. J. Int. Soc. Ultrasound Obstet. Gynecol..

[31] Hides J.A., Miokovic T., Belavý D.L., Stanton W.R., Richardson C.A. (2007). Ultrasound Imaging Assessment of Abdominal Muscle Function During Drawing-in of the Abdominal Wall: An Intrarater Reliability Study. J. Orthop. Sport. Phys. Ther..

[32] Finucane L.M., Downie A., Mercer C., Greenhalgh S.M., Boissonnault W.G., Pool-Goudzwaard A.L., Beneciuk J.M., Leech R.L., Selfe J. (2020). International Framework for Red Flags for Potential Serious Spinal Pathologies. J. Orthop. Sports Phys. Ther..

[33] Maselli F., Palladino M., Barbari V., Storari L., Rossettini G., Testa M. (2022). The diagnostic value of Red Flags in thoracolumbar pain: A systematic review. Disabil. Rehabil..

[34] Chiarello C.M., McAuley J.A. (2013). Concurrent Validity of Calipers and Ultrasound Imaging to Measure Interrecti Distance. J. Orthop. Sport. Phys. Ther..

[35] Wen L., Zhou Q. (2018). Can We Evaluate Hiatal Ballooning by Measuring the Anteroposterior Diameter with 2-Dimensional Translabial Ultrasonography?. J. Ultrasound Med. Off. J. Am. Inst. Ultrasound Med..

[36] Thibault-Gagnon S., McLean L., Goldfinger C., Pukall C., Chamberlain S. (2016). Differences in the Biometry of the Levator Hiatus at Rest, During Contraction, and During Valsalva Maneuver Between Women with and without Provoked Vestibulodynia Assessed by Transperineal Ultrasound Imaging. J. Sex. Med..

[37] Vercellini P., Viganò P., Somigliana E., Fedele L. (2014). Endometriosis: Pathogenesis and treatment. Nat. Rev. Endocrinol..

[38] Greene A.D., Lang S.A., Kendziorski J.A., Sroga-Rios J.M., Herzog T.J., Burns K.A. (2016). Endometriosis: Where are we and where are we going?. Reproduction.

[39] Vercellini P., Buggio L., Frattaruolo M.P., Borghi A., Dridi D., Somigliana E. (2018). Medical treatment of endometriosis-related pain. Best Pract. Res. Clin. Obstet. Gynaecol..

[40] Yong P.J. (2017). Deep Dyspareunia in Endometriosis: A Proposed Framework Based on Pain Mechanisms and Genito-Pelvic Pain Penetration Disorder. Sex. Med. Rev..

[41] Ji R.-R., Nackley A., Huh Y., Terrando N., Maixner W. (2018). Neuroinflammation and Central Sensitization in Chronic and Widespread Pain. Anesthesiology.

[42] López-Ruiz M., Losilla J.M., Monfort J., Portell M., Gutiérrez T., Poca V., Garcia-Fructuoso F., Llorente J., Garcia-Fontanals A., Deus J. (2019). Central sensitization in knee osteoarthritis and fibromyalgia: Beyond depression and anxiety. PLoS ONE.

[43] van Griensven H., Schmid A., Trendafilova T., Low M. (2020). Central Sensitization in Musculoskeletal Pain: Lost in Translation?. J. Orthop. Sports Phys. Ther..

[44] Hides J., Wilson S., Stanton W., McMahon S., Keto H., McMahon K., Bryant M., Richardson C. (2006). An MRI investigation into the function of the transversus abdominis muscle during “drawing-in” of the abdominal wall. Spine.

[45] Urquhart D.M., Hodges P.W., Allen T.J., Story I.H. (2005). Abdominal muscle recruitment during a range of voluntary exercises. Man. Ther..

[46] Dias-Amaral A., Marques-Pinto A. (2018). Female Genito-Pelvic Pain/Penetration Disorder: Review of the Related Factors and Overall Approach. Rev. Bras. Ginecol. Obstet. Rev. Fed. Bras. Soc. Ginecol. Obstet..

[47] Alizadeh A., Farnam F., Raisi F., Parsaeian M. (2019). Prevalence of and Risk Factors for Genito-Pelvic Pain/Penetration Disorder: A Population-Based Study of Iranian Women. J. Sex. Med..

